# The Six-Minute Walk Test in Community-Dwelling Older Adult Women: The Influence of Physical Activity Levels and Age-Related Factors

**DOI:** 10.3390/healthcare13131610

**Published:** 2025-07-04

**Authors:** Rocío Cogollos-de-la-Peña, Gemma Victoria Espí-López, Laura Fuentes-Aparicio, Lucas Monzani, Dagmar Pavlu, Anna Arnal-Gómez

**Affiliations:** 1 Department of Physiotherapy, Faculty of Physiotherapy, University of Valencia, Gascó Oliag St, 5, 46010 Valencia, Spain; rocioinmaculada.cogollos@universidadeuropea.es (R.C.-d.-l.-P.); gemma.espi@uv.es (G.V.E.-L.); anna.arnal@uv.es (A.A.-G.); 2 Department of Physiotherapy, Faculty of Health Science, Universidad Europea de Valencia, Pg. de l’Albereda, 7, El Pla del Real, 46010 Valencia, Spain; 3 Exercise Intervention for Health Research Group (EXINH-RG), Department of Physiotherapy, University of Valencia, 46010 Valencia, Spain; 4 Physiotherapy in Motion, Multispeciality Research Group (PTinMOTION), Department of Physiotherapy, University of Valencia, Gascó Oliag St, 5, 46010 Valencia, Spain; 5 Ivey Business School, Western University, 1255 Western Rd, London, ON N6G 0N1, Canada; lmonzani@ivey.ca; 6 Faculty of Physical Education and Sport, Charles University, 162 52 Prague, Czech Republic; dagmar.pavlu@ftvs.cuni.cz

**Keywords:** women, older adults, Six-Minute Walk Test, physical activity, cardiorespiratory

## Abstract

**Background/Objectives**: In the context of active ageing, functional assessment is key to preserving autonomy in older women. The six-minute walk test (6MWT) is a practical tool for estimating general health, but its results can be influenced by various factors. This study analysed cardiorespiratory variations during the 6MWT in older women according to their physical activity level and age-related variables such as pain, sarcopenia, frailty, and motivation to exercise. **Methods**: A total of 163 older women with musculoskeletal pain, but without cardiac or respiratory conditions, were classified into groups with high (HPA), moderate (MPA), and low (LPA) physical activity. During the 6MWT, heart rate (HR), dyspnoea, and oxygen saturation (SpO_2_) were recorded. Pain, sarcopenia, frailty, and motivation to exercise were also assessed. A repeated-measures multivariate analysis of (co)variance (RM-MANCOVA) was performed. **Results**: The results showed differences in HR depending on the level of physical activity, conditioned by sarcopenia (*p* < 0.05) and walked distance (*p* < 0.001), and in dyspnoea conditioned by pain perception (*p* < 0.01) and social (*p* < 0.001) and psychological (*p* < 0.05) motivation to exercise. There were also differences in SpO_2_ depending on the level of physical activity (*p* < 0.0001). There were differences between the HPA group and both the MPA and LPA group, which had higher HR, higher dyspnoea, and lower SpO_2_ when undergoing the 6MWT test. **Conclusions**: To accurately interpret 6MWT results in older adult women, it is essential to consider physical activity level, perceived pain, sarcopenia, and motivation to exercise, as these factors influence HR, dyspnoea, and SpO_2_. These variables should guide physical activity recommendations for healthy ageing.

## 1. Introduction

The health implications of an ageing population are a topic of growing concern worldwide. The World Health Organization (WHO) has declared 2020–2030 as the healthy ageing decade [1]. Therefore, the WHO emphases the importance of promoting physical activity in older adults as a key strategy to maintain functionality, prevent chronic diseases, and promote quality of life [2]. In this context, healthy ageing is understood as the process of promoting and maintaining functional capacity, which, in turn, allows for well-being in old age [3]. It is an ageing process perspective according to which health should be assessed based on functional capacity, as this is a key indicator for predicting survival and maintaining a good quality of life [4]. Yet, healthy ageing is not without its challenges, given that ageing is a complex, multifactorial process in which changes occur in all physiological systems.

With regard to sex, prior studies have identified differences among men and women in their ageing process. Sex-based differences in longevity, mortality, and age-related diseases are well-documented in the literature [5,6,7]. In developed countries, females generally live longer than males and have significantly lower mortality rates at all ages. However, it is also known that women experience higher levels of morbidity compared to men [5]. Women’s health and well-being are influenced by biological, social, and environmental factors. In older adult women, reduced estrogenic production impacts bone density, muscle mass, and fat distribution, affecting functional capacity and chronic disease risk [8]. The hormonal changes during menopause aggravate conditions such as sarcopenia and osteoporosis, as well as cardiorespiratory disorders, which hinders physical activity and independence [9,10].

Musculoskeletal pain, prevalent in up to 80% of older adults, significantly affects quality of life, mood, physical capacity, and social relationships [11]. Furthermore, non-specific (exclusion of specific causes by means of anamnesis) [12] neck and low back pain are the leading causes of global disability [13]. Specifically, regarding the cervical area, the trapezius is one of the neck muscles where pain in older adults is most prevalent [14]. Moreover, women experience more chronic pain than men, defined as pain lasting more than 3 months [15]; this may be due to hormonal differences, which affect muscle strength and gait [16].

In addition to physiological factors, psychosocial factors influence healthy ageing in women. Older women are often less physically active due to cultural stereotypes of gender roles, which affects their ability to age healthily and increases their risk of disease [17]. Promoting physical activity can help to overcome these barriers as physical activity benefits women of all menopausal statuses [18]. Among psychosocial factors, motivation to exercise is crucial for increasing activity, which in turn is essential for healthy behaviours. Motivation is understood as a person’s willingness to act in a certain way based on a conglomerate of physical, social, and psychological factors occurring at a given time [19]. Motivation is related to neurocognitive mechanisms such as the activation of analgesia and dopaminergic reward systems, which could influence the performance of physical activity [20]. However, there is often a gap between the intention and the performance of physical activity [21].

Moreover, physical activity has become an important topic in relation to the ageing process. Thus, regular practice is related to prevention and treatment of most chronic diseases and to a reduction in premature mortality [2,9]. In addition, physical activity has been shown to have a significant effect on mental and cognitive health, sleep quality, cognitive performance, prevention of falls and fractures, and improvement of functional capacity in older adults [22]. The WHO recommends that adults aged 65 and older should practice physical activity for 150 min per week [9].

Given the importance of physical activity for healthy ageing in women, assessing their functional capacity is crucial. In the last decade, many submaximal tests have been developed as an alternative to maximal stress tests [23]. Most of them propose “walking” as the main test, as it is a good indicator for older adults [24]. Some functional capacity tests are the stair climbing test and the shuttle test or “Incremental Shuttle Walking Test”, but the six-minute walk test is the most widely used, as it is the best known by professionals, easy to perform, easy to interpret, and sustainable [25,26]. The six-minute walk test (6MWT) is a reliable tool for evaluating the integrated respiratory, cardiovascular, and musculoskeletal response to exercise stress [26,27]. Its simplicity, safety, low cost, and similarity to daily activity make it ideal for clinical and research environments [28]. It was designed to assess functional capacity and exercise tolerance in older adults and those with cardiopulmonary conditions [27]. However, its use has extended to various health problems and healthy individuals [29,30,31].

Prior studies investigating the use of the 6MWT focused either on populations with cardiac or respiratory pathology or on comparing older adults of both sexes, finding sex-based differences in the results of several studies [31,32,33,34]. Nevertheless, the previous literature has stated that few studies have focused exclusively on women [29], meaning that there is poor understanding of the specific applicability of the 6MWT to healthy older women. This needs to be considered, as women do not age in the same way as men due to hormonal and physiological factors. The study by Steffens et al. [29] evaluates the influence of physical activity on the results of the 6MWT and concludes that the level of physical activity influences the distance walked; however, age and gender were not considered as specific factors, so, as noted by Bautmans et al. [34], this could affect the interpretation of the results. The 6MWT test offers several advantages for the assessment of training capacity in older people, and a more specific and gender-differentiated approach would promote more effective training.

The 6MWT is a valid functional test for older adult women due to its safety, similarity to daily life, and reliability. Therefore, this study aims to explore the association between physical activity levels and results related to heart rate (HR), dyspnoea, and oxygen saturation (SpO_2_) in older adult women when performing the 6MWT. We anticipate differences in HR, dyspnoea, and SpO_2_ before and after the test, influenced by physical activity levels, pain, sarcopenia, frailty, and motivation to exercise.

The 6MWT is a valid functional test for older adult women due to its safety, similarity to activities of daily life, and reliability. It is important to include other clinical variables. In this sense, the hypothesis of this study is that, in addition to physical activity levels, there are other relevant variables that should be considered for a more accurate interpretation of the cardiorespiratory results obtained in the six-minute walk test.

Therefore, the aim of our study is to analyse the association between physical activity levels and cardiorespiratory outcomes such as heart rate (HR), dyspnoea, and oxygen saturation (SpO_2_) during performance of the 6MWT, incorporating other variables that are usually less considered, such as pain, sarcopenia, frailty, and motivation towards exercise.

## 2. Materials and Methods

### 2.1. Study Design

This was a cross-sectional observational study conducted in the Laboratories of the Faculty of Physiotherapy of the University of Valencia from October 2021 to May 2022. Participants were informed beforehand and signed a written informed consent form before participating in the study. The study was approved by the Human Ethics Committee for Research of the University of Valencia (No. 1393203) and registered, as part of a larger study, in the ClinicalTrials.gov database (identifier: NCT04345211). The manuscript was drafted according to the Strengthening the Reporting of Observational Studies in Epidemiology (STROBE) protocol.

### 2.2. Participants

For the recruitment of the sample, we contacted associations of older adults by telephone and social networks. The present study included women who met the following inclusion criteria: aged 60–80 years old, with non-specific musculoskeletal or osteoarticular chronic pain (>3 months), non-smokers (>6 months). The following exclusion criteria were considered: women with a history of respiratory or cardiac condition, with an inability to walk without technical aids or assistance, with acute musculoskeletal or osteoarticular pain at the time of assessment, and with cognitive impairment (Mini-Mental State Examination < 25 points).

Physical activity levels were determined by asking the participants about the frequency and intensity of their weekly activity, following the WHO guidelines [35] and previous studies in the literature [17]. A researcher blinded to the study objectives and not involved in the assessments asked the participants by phone about their physical activity habits: whether they currently practiced exercise (yes/no), and if so, about the frequency of the exercise (1 day/week; 2–3 days/week; 5 days/week; 7 days/week). Then, they were asked about the intensity of their physical activity (moderate: physical activity that makes them breathe a little harder than normal whilst maintaining an uninterrupted conversation; or vigorous: activity which requires a strong physical effort and makes them breathe much harder than normal, meaning they are not able to maintain an uninterrupted conversation) [36] and the time spent (minutes/week) at these intensities, in order to classify them into three different physical activity levels [35]:

- Low physical activity (LPA): those who performed less than 150 min of moderate aerobic physical activity per week, or some form of vigorous aerobic physical activity for 75 min.
- Moderate physical activity (MPA): those who performed between 150 and 300 min of moderate aerobic physical activity per week, or some form of vigorous aerobic physical activity for 150 min per week.
- High physical activity (HPA): those who performed more than 300 min per week of moderate aerobic physical activity, or more than 150 min per week of vigorous aerobic physical activity.

### 2.3. Procedure

A physiotherapist with 15 years of experience in treating musculoskeletal disorders in older people conducted individual face-to-face assessments. Before the session, procedures were explained in detail to all participants. An extensive anamnesis covering sociodemographic, anthropometric, and clinical data was conducted, followed by the 6MWT to assess study variables.

Sociodemographic data included age, marital status, educational level, and employment status. Anthropometric and clinical data included body weight using a Tanita BC 601 (TANITA Ltd., Tokyo, Japan), height with a SECA 213 stadiometer (Seca Ltd., Hamburg, Germany), body mass index (BMI) calculation (kg/m^2^), medical history of hypertension or cholesterol, and comorbidities using the Spanish version of the Abbreviated Charlson Comorbidity Index.

### 2.4. Outcome Measures

- Musculoskeletal pain: The pressure pain threshold (PPT) in trapezius muscles was measured by the minimal pressure (kg/cm^2^) which induces pain by pressure algometry (Wagner Instruments FDK 20). The patient was seated, with her back straight and supported by a chair, completely relaxed, and the trapezius muscles were assessed bilaterally by taking three measurements on each side, with a thirty-second rest period in between them. The exact location was in the middle part of the anterior border of the upper trapezius, in the most vertical fibres that insert in front of the clavicle (upper trapezius muscle). We applied the tip of the algometer perpendicular to the muscle and maintained a pressure at a gradual rate of 1–2 kg/cm^2^ per second to avoid abrupt reactions and ensure an accurate measurement of the pain threshold. Subjects were instructed to make a signal at the moment they experienced pain, in order to have an accurate recording [37]. For the analysis, the mean of three scores on both sides was obtained. CCI = 0.91 (IC del 95%: 0.82; 0.97) [38].
- Perceived pain: The Visual Analogue Scale (VAS) was used. Non-specific perceived pain was assessed in the following areas: head and neck (VAS-HN), shoulders (VAS-S), dorsal area (VAS-D), lumbar area (VAS-L), and pelvic and hip area (VAS-PH). The VAS is a one-dimensional measure of pain intensity which has been widely used in various adult populations. The VAS is a continuous scale composed of a horizontal or vertical line, typically 10 centimetres long (100 mm). The respondent was asked to indicate their pain intensity with a score of 0 being “no pain” and 10 being the “worst pain imaginable”, namely, no pain (0–4 mm), mild pain (5–44 mm), moderate pain (45–74 mm), and severe pain (75–100 mm). It has shown been to be reliable (ICC = 0.810) [39].
- Sarcopenia screening: The Spanish version of the SARC-F questionnaire was used to screen sarcopenia. It consists of 5 components: strength, assistance with walking, rising from a chair, climbing stairs, and falls. SARC-F scores range from 0 to 10, with 0 to 2 points for each component. A score ≥ 4 predicts sarcopenia (Rho = 0.43 to 0.76; CCI = 80; sensitivity =70.63%; specificity= 78.67%) [40].
- Frailty: The Spanish version of the 5-item FRAIL questionnaire was used to assess frailty screening. It has 5 components: fatigue, endurance, ambulation, illness, and weight loss. Frailty scores range from 0 to 5 (i.e., 1 point for each component; 0 = best to 5 = worst) and total score represents frail (3–5), pre-frail (1–2), and robust (0) health status [1] (rho = 0.41–0.74; CCI = 0.82, validity 9.6%) [41].
- Motivation to exercise: This was measured using the validated version of the Exercise Motivation Index (EMI). The questionnaire consists of 15 statements followed by a five-point rating scale for each statement, ranging from 0 (not at all true for me) to 4 (very true for me). Three sub-scores are calculated for physical, psychological, and social motivation by summing the scores in each one and dividing them by the number of statements in each area. Previous evidence has shown the reliability and validity of this index [42].
- Functional capacity: This was assessed using the six-minute walk test (6MWT). The aim of this test is to walk the furthest distance possible in 6 min. Participants were asked to wear comfortable clothes and footwear, and they were instructed not to eat large meals or drink sugary or caffeinated beverages immediately before the test. For the test, they walked between two cones, which marked the turning point, in a 30-metre corridor. They were instructed to try to cover as much distance as possible within six minutes without running. They could slow down or stop and rest if necessary and resume walking as soon as possible. The physiotherapist demonstrated the walking lap herself. The 6MWT was performed inside the aforementioned university, in an enclosed corridor with a hard surface [27]. Before (pre-test) and after (post-test), the following variables were assessed:
  - Heart rate was assessed using a finger pulse oximeter (Finger Pulse Oximeter FS20C with OLED display). Participants stayed seated and relaxed while the pulse oximeter was positioned on their index finger, ensuring it was clean and devoid of nail polish. Once the pulse oximeter was in place and after a few seconds for it to detect the pulse, the HR data was obtained. HR was measured immediately after the test and then, for safety reasons, a few minutes later to check that they had recovered their initial HR [28]. The normal resting HR of healthy women is on average 80 bpm (range: 78–82 bpm) [43].
  - Degree of dyspnoea: Dyspnoea is a subjective sensation of suffocation, which was quantified using the modified Borg scale, this being a numerical scale from 0 to 10 (0: total absence of dyspnoea; 1–2: very slight dyspnoea; 3–4: mild dyspnoea; 5–6: moderate dyspnoea; 7–8: severe dyspnoea; 9–10: maximum dyspnoea) [44]. Participants were shown the scale so that they could indicate their degree of dyspnoea before and after the test.
  - Oxygen saturation was assessed with the same device and procedure as HR. After a brief pause, the pulse oximeter registered the pulse and determined the SpO_2_ level. Typically, SpO_2_ levels between 96% and 100% are considered normal at sea level [45].

During the test, the women had the pulse oximeter on their finger to control the mentioned variables. The 6MWT distance (6MWT-D) was the total number of metres walked at the end of the test, and it was recorded. The 6MWT is a reliable measure (ICC = 0.82–0.99) [46].

### 2.5. Sample Size Calculation

The target sample size was calculated using G*Power 3.1.9.7. An “a priori” power analysis for three independent groups was conducted, aiming to detect small-to-moderate effect sizes (Cohen’s d = 0.25) with α = 0.05 and 1 − β = 0.80. The results indicated a minimum sample size of 159 participants to achieve sufficient statistical power.

### 2.6. Statistical Analysis

All statistical analyses were performed using SPSS 27 (SPSS Inc., Chicago, IL, USA). Continuous variables were described as means and standard deviations (SDs), while categorical variables were described as frequencies and percentages. The Shapiro–Wilk test was used to assess normality. ANOVA compared sociodemographic and clinical characteristics across physical activity levels for continuous variables and the chi-squared test for categorical variables.

The level of significance was set at *p* < 0.05 (α = 0.05; 1 − β = 0.80). Pearson’s r was calculated to assess correlations between dependent variables (HR, dyspnoea, SpO_2_, 6MWT-D, PPT, VAS for all areas, sarcopenia screening, frailty, and the three facets of motivation to exercise). A repeated-measures multivariate analysis of (co)variance (RM-MANCOVA) was used to achieve the study aim [47]. Bonferroni’s correction was used for pairwise comparisons. (See Supplementary Material SI for a more detailed explanation and robustness checks.)

The general linear model included HR, dyspnoea, and SpO_2_ as dependent variables. Scores in the 6MWT-D, PPT, VAS-HN, VAS-S, VAS-D, VAS-L, VAS-PH, SARC-F, FRAIL, and EMI were covariates. Physical activity level was a between-subject predictor, and time elapsed between measurements was a within-subject factor. An interaction term tested whether variations in measurements were influenced by physical activity level. For RM-MANCOVA results, a non-significant Box’s M test ensures model trustworthiness. Significant *p*-values for Wilks’ Lambda or the F-test indicate predictor effects on dependent variables. A significant between–within interaction term suggests that elapsed time effects on dependent variables vary across physical activity levels. Partial Eta squared (partial η2) can be converted into Cohen’s d effect sizes.

## 3. Results

Of the 184 participants initially recruited, 163 were included in this study, as 10 participants were excluded for not meeting the inclusion criteria and 11 declined to participate for personal reasons. Of these 163 people, 51 women had a low activity level (LPA), 48 had a moderate activity level (MPA), and 64 had a high activity level (HPA). The analysed sample had a mean age of 69.41 years (SD 5.1); 47.2% were married women, and 61.3% had a university degree. The HPA group showed significant differences with the other two groups regarding education level. The mean BMI was overweight (26.32; SD 4.87), but the participants did not have high values of comorbidity, hypertension, or cholesterol (Table 1).

Regarding physical activity levels, the three groups showed similar pain perception and PPT values, although both pain variables showed a better score trend (less pain) for the MPA and HPA group than the LPA. The FRAIL questionnaire showed a mean score of 0.27 (SD 0.52) for the total sample, and pre-frailty status was homogeneous in the three groups, with the HPA group showing a higher percentage of “no frailty” (0–2). However, the screening for sarcopenia was only positive for some participants in the LPA group, and the mean score for the total sample was 0.79 (SD 0.89) points. The physical characteristics of the participants distributed by physical activity level are depicted in Table 2.

### RM-MANCOVA

After controlling for musculoskeletal pain, sarcopenia, frailty, and motivation to exercise, we detected a medium-to-large effect of PA level on HR (F (1, 148) = 9.64, *p* < 0.0001; partial η^2^ = 0.12; Cohen’s d = 0.72). More precisely, our between–within-subjects post hoc tests showed that participants with LPA level reported a significantly higher HR than those with HPA level before and after the test (Pre–Post = −16.60; SE = 3.32; *p* < 0.001; 95% CI = [−24.20, −8.13]). Similarly, participants with an MPA level had a significantly higher HR than those who reported a HPA level (Pre–Post = −14.61; SE = 3.35; *p* < 0.001; 95% CI = [−22.73, −6.49]). In other words, PA level significantly impacts HR, and this effect is significantly influenced by 6MWT-D and a positive screening for sarcopenia. Moreover, there are differences between the HPA group and both the MPA and LPA groups, with the latter two groups showing higher levels of HR during the 6MWT. 

After controlling for the above-mentioned covariates, our model also detected a medium-to-large effect of PA level on dyspnoea (F (1, 148) = 8.50, *p* < 0.0001; partial η^2^ = 0.10; Cohen’s d = 0.68). More precisely, our between-subjects post hoc tests showed that LPA-level participants reported a significantly higher dyspnoea than those with HPA level (Pre–Post = −0.80; SE = 0.20; *p* < 0.001; 95% CI = [−1.28, −0.33]). Similarly, MPA-level participants showed higher scores in dyspnoea in the post-test than those with HPA level. Despite being in the expected direction, the estimated mean differences between participants with HPA and MPA levels did not reach statistical significance at the *p* < 0.05 level but did so at the *p* < 0.10 level. More precisely, our between-subjects post hoc tests show that participants with HPA level showed a significantly higher SpO_2_ than those with LPA level (Pre–Post = 1.04; SE = 0.29; *p* < 0.001; 95% CI = [0.34, 1.74]). Similarly, HPA-level participants had significantly higher SaO_2_ than those who reported an MPA level (Pre–Post = 1.00; SE = 0.30; *p* < 0.001; 95% CI = [0.28, 1.71]).

In other words, the PA level of older adult women significantly impacts their HR, dyspnoea, and SpO_2_ when performing the 6MWT. These effects were robust after controlling for the effects of 6MWT-D and sarcopenia on HR and those of PPT and exercise motivation on dyspnoea. Moreover, there were differences between the HPA-level group and both the MPA and LPA level, both of which had higher HR, higher dyspnoea, and lower SpO_2_ when undergoing the 6MWT test.

Our within-subjects factor (elapsed time) did not have a main effect on HR, dyspnoea, or SpO_2_ (Pillai’s trace = 0.03; F (3, 146) = 1.34; ns; partial η^2^ = 0.03). However, a medium-to-large, significant between–within interaction effect (Pillai’s trace = 0.26; F (6, 292) = 7.18; *p* < 0.0001; partial η^2^ = 0.13; Cohen’s d = 0.77) was detected. In other words, the estimated marginal means for HR, dyspnoea, and SpO_2_ varied significantly between pre-test and post-test measurements and across participant groups (LPA, MPA, HPA).

**Figure 1:**
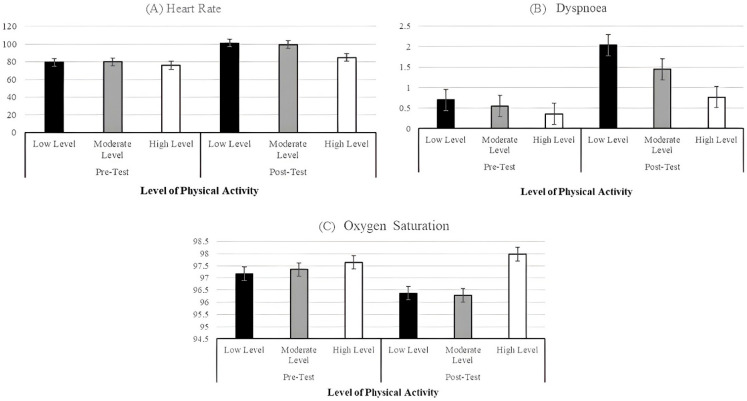
Estimated marginal means illustrating the effect of level of physical activity x time on heart rate (**A**), dyspnoea (**B**) and SPO_2_ (**C**).

Table 3 and Figure 1 show the estimated marginal means for HR, dyspnoea, and SpO_2_. For participants who reported an LPA level, their HR increased by 27.62% between pre- and post-test measurements, their dyspnoea increased by 191%, and their SpO_2_ was reduced by 0.01%. For those participants who reported an MPA level, their HR increased by 24.40%, their dyspnoea score increased by 164%, and their SpO_2_ was reduced by 0.01%. Finally, in participants who reported an HPA level, the increase in HR was 11.75%; for dyspnoea, the increase was 139%; and SaO_2_ was increased by +0.03%.

## 4. Discussion

The present study reveals that there are cardiorespiratory differences in older adult women in the 6MWT test. Older adult women’s level of physical activity was significantly related to variability in cardiorespiratory performance during the 6MWT. Factors such as pain, sarcopenia, and motivation to exercise influenced HR, dyspnoea, and SpO_2_ values before and after the test. Changes in HR before and after the 6MWT are directly related to walked distance and sarcopenia. Dyspnoea was associated with the pressure pain threshold in the trapezius, as well as with social and psychological motivation to exercise. However, SpO_2_ was influenced by the level of physical activity but not by other individual characteristics. Our findings provide support for our hypothesis; thus, there are variations in test performance according to the level of physical activity and other age-related variables.

The study by Steffens et al. [29] concluded that the level of physical activity influences the distance walked, but they did not take into account how other variables specific to age or women may have affected this performance. Therefore, our study is the first to investigate cardiorespiratory performance in the 6MWT in older women without cardiac or respiratory pathology according to their physical activity levels and considering other characteristics such as pain, frailty, sarcopenia, or motivation to exercise. Understanding these variations can help health professionals to interpret the 6MWT more holistically, considering that there are physiological and psychosocial factors that influence the 6MWT results. We caution health professionals to take these associations into account when interpreting the results of the 6MWT, as the 6MWT is widely used to assess exercise capacity in various diseases [48].

Our results showed that physical activity levels influence the HR of older adult women measured before and after the 6MWT. Those who were physically active (more than 300 min/week, HPA), showed a lower HR before and after the test. As described by Cordero et al. [49], the cardiovascular adaptations caused by high levels of physical activity decrease resting HR and lead to better cardiovascular adaptation to exertion [49]. Our results, together with those reported Stefferns et al. [29], reinforce the idea of considering the levels of physical activity to interpret the cardiovascular results of the 6MWT test; being highly active leads to a better cardiovascular and functional situation, but there are other factors specific to age and women that should also be taken into account and that affect both the levels of activity and HR.

The results regarding sarcopenia screening showed that this age-related variable interfered with HR. SARC-F introduces the assessment and treatment of sarcopenia into clinical practice, since it is an inexpensive and convenient method for sarcopenia risk screening [50]. Detecting sarcopenia in older women is of paramount importance since it is understood as the loss of strength and muscle mass, which can affect functional capacity [50]. In the present study, women with positive screening for sarcopenia were those who performed less physical activity and showed a higher HR than those in the HPA group. According to the review by Dominguez-Chavez et al. [51], in terms of lifestyle factors related to sarcopenia, performing at least one hour of heavy physical activity more than three days per week, which is the PA level of our study’s HPA group, decreases the probability of developing sarcopenia. The positive screening for sarcopenia in the LPA and MPA groups may be due to the fact that the decrease in strength and muscle mass makes it more difficult to perform physical activity [52], and this, in turn, leads to a lower cardiovascular efficiency, conditioned by the level of activity [53]. Therefore, it is not only high levels of physical activity that may influence HR but also the presence of sarcopenia, as well as inactivity, which, in turn, accelerates sarcopenia [54]. Bearing in mind that women have higher levels of sarcopenia than men [55], it is justified that, when interpreting the results of the 6MWT, other factors or characteristics that may condition these results should be taken into account.

With regard to the distance walked, as noted by Steffens et al., activity level is a good predictor of distance walked, as are age and BMI [29]. In our study, women with MPA were the group that walked more metres, although all groups showed a walked distance within the normal values according to the reference equations [33]. It should be noted that the MPA group was the tallest group, and as shown by Camarri et al. (2006) [30] in their study, height, but not leg length, is a significant predictor of distance walked. In contrast, the study by Joobeur et al. [56] showed that taller people have a longer stride and therefore also cover more metres.

Regarding dyspnoea, our findings revealed that the level of physical activity significantly influences its intensity. Women who performed less physical activity (LPA) had higher dyspnoea values both before and after the test. In the study by Acosta et al. [57], a moderate exercise intervention was conducted 3 days a week and a significant decrease in dyspnoea was found. These results are also supported by a previous study by Lacasse et al. [58] and are consistent with our study, according to which women who belonged to the HPA group had lower levels of dyspnoea. We can thus establish that, indeed, the level of physical activity influences the intensity of dyspnoea. On the other hand, there are other factors which can affect dyspnoea, such as perceived pain, as shown in our study. According to Awadallah et al. [59], patients with neck pain may present limited neck movement, resulting in limited pulmonary function, in turn leading to increase breathing effort and thus higher levels of dyspnoea. In our study, women with the lowest tolerance to trapezius pain coincided with the highest levels of dyspnoea both before and after the test and were also the least physically active.

Dyspnoea was also affected by social and psychological motivational factors. Previous research has shown that motivation to exercise in older adult women tends to prioritise meeting other people of the same age, satisfying their basic psychological need to relate to others, which favours the practice of physical activity [17]. In line with our study, Renzi et al. showed that social and psychological motivation prevail over physical motivation to exercise [60]. Therefore, healthcare professionals should take into consideration that if social and psychological motivation are encouraged, more physical activity may be practiced and this, in turn, promotes better functional capacity with lower values of dyspnoea; more specifically, moderate or vigorous physical activity helps to reduce dyspnoea [61]. This may explain why, in our study, the LPA group had higher values of dyspnoea in the 6MWT.

In terms of SpO_2,_ normative levels in older female athletes range from 95% to 97%, with a 2% variability. Rates below 90% at rest may indicate adverse medical conditions and impaired respiratory function [62]. The total sample of older adult women in our study had SpO_2_ levels at 97% before the test, but after the test, those with HPA remained within the same SpO_2_ level or even slightly improved, while those with LPA or MPA dropped to 96%. This saturation drop is considered normal since previous research has stated that with great physical effort, arterial saturation drops can happen [63]. Our study has now revealed that depending on the level of previous physical activity, these values can change. This is consistent with Miyachi and Katayama’s study [64] examining the relationship between vigorous exercise and changes in SpO_2_, which found that people with lower aerobic fitness experienced a significant decrease in SpO_2_ when performing high-intensity physical activities [64]. Barbosa et al. also showed that in women over 65 years of age performing the 6MWT, there was a significant relationship between lower physical activity and higher O_2_ cost [65].

Other studies have investigated which characteristics may be predictors of 6MWT distance in healthy older adults [33,48]. In the study by Steffen et al. (2013) [29] conducted in healthy older adult women, the authors investigated how physical activity levels can predict distance in the 6MWT, only comparing physically active women with sedentary ones and taking into account anthropometric and lung function values. However, in the present study, we divided the sample into three levels of physical activity and considered, in addition to anthropometric characteristics, other age-related variables such as pain, sarcopenia, frailty, or motivation to exercise, all of which showed statistically significant differences, except for frailty.

The present study is not without some limitations. The 6MWT is a simple, inexpensive, and safe test that corresponds to the activities of daily living of the older adult population, but as it is a self-paced test, in which each participant is motivated by verbal stimuli, the participant’s effort or speed in covering the distance (not measured in our study) may be a limitation for assessing HR, dyspnoea, and SpO_2_ parameters. Another limitation of the study could be the classification of activity levels, which was carried out through a self-reported questionnaire in which women were asked about their frequency and type of physical activity, as well as their training intensity, and not with a validated questionnaire such as IPAQ, although it was based on the previous scientific literature. Moreover, when assessing sarcopenia, all of the EWGSOP-2 diagnostic criteria could have been followed, instead of only the SARC-F. However, the EWGSOP2 recommends use of the SARC-F questionnaire as a way to elicit self-reports from patients on signs that are characteristic of sarcopenia [50]. In addition, given the multifactorial nature of pain and its potential associations with depression, anxiety, and low self-efficacy, these variables could have been included to analyse their impact on physical performance. Finally, the age range in our study was 60 to 80 years, and given that the population has an increasingly higher life expectancy, for future research, it would be desirable to extend the age limit above 80 years.

Despite the limitations, this study investigates the relationship of physical activity levels and other age-related characteristics that may influence 6MWT results in older women without cardiac or respiratory pathology. Therefore, a correct interpretation of the 6MWT results taking into account the specific characteristics of each woman can help to design more effective and adapted therapeutic exercise programmes.

## 5. Conclusions

To accurately interpret the cardiorespiratory results of the 6MWT, the level of physical activity and other variables such as perceived pain, sarcopenia, and both social and psychological motivation to exercise, which influence HR, dyspnoea, and SpO_2_ results, must be taken into account.

These variables should be considered if the 6MWT is used as a functional test to recommend physical activity for healthy ageing in older women.

## Figures and Tables

**Table 1:** Sociodemographic and anthropometric characteristics of the participants distributed by their level of physical activity.

Outcomes	Categories/ Units	Total Sample n = 163	Low Activity (LPA) n = 51	Moderate Activity (MPA) n = 48	High Activity (HPA) n = 64	*p*
Age (mean/SD)	Years	69.41 (5.13)	69.63 (5.40)	69.92 (4.81)	68.86 (5.17)	0.526
Marital Status n (%)	Single	21 (12.90%)	9 (17.60%)	5 (10.40%)	7 (10.90%)	0.403 ^B^
	Married	77 (47.20%)	19(37.30%)	29 (60.40%)	29 (45.30%)	
	Widowed	36 (22.10%)	14 (27.50%)	7 (14.60%)	15 (23.40%)	
	Divorced/ Separated	26 (15.90%)	8 (15.70%)	7 (14.60%)	11 (17.20%)	
Level of education n (%)	High school	62 (38%)	23 (45.10%)	23 (48%)	16 (25%)	0.065 ^B^
	University degree	100 (61.3%)	28 (54.90%)	25 (52.10%)	47 (73.40%)	
Weight (mean/SD)	kg	67.16 (11,99)	69.37 ^£^ (13.12)	69.90 * (12.45)	63.35 *^£^ (9.64)	0.004 ^A^
Height (mean/SD)	m	1.59 (0.74)	1.59 (0.07)	1.62 (0.08)	1.58 (0.06)	0.031 ^A^
Body mass index (mean/SD)	Kg/m^2^	26.32 (4.87)	27.21 (5.50)	26.67 (4.90)	25.33 (4.18)	0.100 ^A^
Charlson index (mean/SD)	Total score	0.28 (0.67)	0.41 (0.80)	0.31 (0.68)	0.14 (0.53)	0.093 ^A^
Charlson index n (%)	No comorbidity	148 (90.80%)	45 (88.20%)	42 (87.50%)	61 (95.30%)	N.A 0.098 ^B^
	Low comorbidity	12 (7.40%)	4 (7.80%)	6 (12.50%)	2 (3.10%)	
	High comorbidity	3 (1.80%)	2 (3.90%)	0	1 (1.60%)	
Blood pressure n (%)	Normotensive	120 (73.60%)	34 (66.70%)	33 (68.80%)	53 (82.80%)	
	Hypertensive	43 (26.40%)	17 (33.30%)	15 (31.30%)	11 (17.20%)	
Cholesterol n (%)	Normolipidaemia	96 (58.90%)	32 (62.70%)	27 (56.30%)	37 (57.80%)	0.786 ^B^
	Dyslipidaemia	67 (41.10%)	19 (37.30%)	21 (43.80%)	27 (42.20%)	

Note: ^A^: Anova; ^B^: chi-square; *: *p* < 0.05 between MPA and HPA; ^£^: *p* < 0.05 between LPA and HPA; N.A: not applicable.

**Table 2:** Physical characteristics of participants distributed by physical activity level. Means, standard deviations, and percentages.

Outcomes	Categories/ Units	Total Sample n = 163	Low Activity (LPA) n = 51	Moderate Activity (MPA) n = 48	High Activity (HPA) n = 64	*p*
PPT (mean/SD)	Kg/m^2^	3.44 (1.37)	3.1 (.97)	3.6 (1.43)	3.54 (1.58)	0.28 ^A^
VAS (mean/SD)	0–10					
	Head/neck	2.34 (2.70)	2.63 (3.14)	2.42 (2.53)	2.06 (2.61)	0.54 ^A^
	Shoulder	1.79 (2.79)	2.31 (3.22)	1.35 (2.27)	1.69 (2.74)	0.22 ^A^
	Dorsal	1.26 (2.18)	1.39 (2.43)	0.75 (1.73)	1.53 (2.25)	0.15 ^A^
	Lower back	3.67 (3.14)	3.14 (3.04)	3.88 (3.10)	3.95 (3.23)	0.34 ^A^
	Pelvis/hip	2.17 (3.05)	1.96 (2.60)	1.73 (3.05)	2.67 (3.22)	0.22 ^A^
Frailty n (%)	Pre-frailty (>2)	38 (23.3%)	13 (25.5%)	9(18.8%)	16 (25%)	
	No frailty (0–2)	125 (76.7%)	38 (74.5%)	39(81.3%)	48 (75%)	0.80 ^B^
Sarcopenia n (%)	Yes (>4)	2(1.2%)	2 (3.9%)	0	0	N.A.
	No (0–4)	161(98.8%)	49 (96.1%)	48 (100%)	64 (100%)	
Motivation (mean/SD)	0–4					
	Physical	3.45 (0.64)	3.46 (0.71)	3.47 (0.64)	3.42 (0.58)	0.92 ^A^
	Social	2.93 (0.97)	3.01 (0.99)	3.05 (0.91)	2.78 (0.98)	0.26 ^A^
	Psychological	3.24 (0.63)	3.25 (0.65)	3.23 (0.54)	3.24 (0.68)	0.99 ^A^
6MWT-D (mean/SD)	metres	506.49 (66.60)	501.92 (54.19)	523.13 (78.11)	497.66 (64.93)	0.12 ^A^
HR pre (mean/SD)	bpm	78.08 (12.08)	78.55 (9.98)	81.27 (14.2)	75.31 (11.39) *^£^	0.03 ^A^
HR post (mean/SD)	bpm	94.17 (21.06)	100.29 (21.92)	102.21 (21.28)	83.25(14.86) *^£^	0.00 ^A^
SpO_2_ pre (mean/SD)	%	97.40 (1.35)	97.16 (1.18)	97.35 (1.63)	97.64 (1.23)	0.16 ^A^
SpO_2_ post (mean/SD)	%	96.97 (2.42)	96.41 (1.96)	96.21 (3.43)	97.98 (1.24) *^£^	0.00 ^A^
Dyspnoea pre (mean/SD)	(0–10)	0.52 (0.81)	0.75 (0.71)	0.56 (0.54)	0.31 (1.00) ^£^	0.02 ^A^
Dyspnoea post (mean/SD)	(0–10)	1.37 (1.70)	2.18 (1.90)	1.44 (1.10)	0.67 (1.40) ^£^	0.00 ^A^

Note: ^A^: Anova; ^B^: chi-square; *: *p* < 0.05 between MPA and HPA; ^£^: *p* < 0.05 between LPA and HPA; N.A.: not applicable; PPT = pressure pain threshold; VAS = Visual Analogue Scale; 6MWT-D: 6 min walk distance; HR = heart rate; SpO_2_ = oxygen saturation.

**Table 3:** Estimated marginal means for the effect of physical activity levels on heart rate, dyspnoea, and oxygen saturation.

	PA Level	Pre (i)			Post (J)			Δ% I − J	Post Hoc Comparisons		
HR		EMM	SE	95% CI	EMM	SE	95% CI		I − J	SE	95% CI
	LPA	79.18	1.69	[75.83, 82.52]	101.05	2.43	[96.24, 105.86]	+27.62%	−16.76 ***	1.04	[−18.81, −14.72]
	MPA	79.85	1.75	[76.40, 83.31]	99.34	2.51	[94.37, 104.30]	+24.40%			
	HPA	75.88	1.51	[72.90, 78.85]	84.80	2.16	[80.53, 89.08]	+11.75%			
D	LPA	0.70	0.11	[0.49, 0.92]	2.04	0.22	[1.60, 2.48]	+191%	−0.89 ***	0.11	[−1.10, −0.67]
	MPA	0.55	0.11	[0.32, 0.77]	1.45	0.11	[1.00, 1.91]	+164%			
	HPA	0.36	0.10	[0.17, 0.55]	0.77	0.20	[0.38, 1.16]	+139%			
SpO_2_	LPA	97.17	0.20	[96.78, 97.56]	96.37	0.33	[95.73, 97.02]	−0.01%	0.51 **	0.18	[0.16, 1.10]
	MPA	97.34	0.20	[96.93, 97.74]	96.28	0.34	[95.61, 96.94]	−0.01%			
	HPA	97.64	0.18	[97.30, 97.99]	97.97	0.29	[97.39, 98.53]	+0.03%			

Note: *** *p* < 0.001; ** *p* < 0.05; HR = heart rate; D = dyspnoea; SpO_2_ = oxygen saturation; LPA = less than 150 min a week; MPA = up to 150 min a week; HPA = up to 300 min a week; estimated marginal means, SE, and 95% CI.

## Data Availability

Data is available upon reasonable request.

## Supplementary Materials

The following supporting information can be downloaded at https://www.mdpi.com/article/10.3390/healthcare13131610/s1. Supplementary Material SI: Statistical Analysis and Robustness checks

## Abbreviations

The following abbreviations are used in this manuscript:

6MWT	Six-Minute Walk Test
HPA	High Physical Activity
MPA	Moderate Physical Activity
LPA	Low Physical Activity
HR	Heart Rate
SpO_2_	Oxygen Saturation
WHO	World Health Organization
PPT	Pressure Pain Threshold
VAS	Visual Analogue Scale
VAS-HN	Visual Analogue Scale head and neck
VAS-S	Visual Analogue Scale shoulders
VAS-D	Visual Analogue Scale dorsal area
VAS-L	Visual Analogue Scale lumbar area
VAS-PH	Visual Analogue Scale pelvic and hip area
EMI	Exercise Motivation Index
6MWT-D	6MWT Distance
