# Supplementary material for: The Six-Minute Walk Test in Community-Dwelling Older Adult Women: The Influence of Physical Activity Levels and Age-Related Factors
Source: Healthcare (Basel). 2025 Jul 4;13(13):1610. doi: 10.3390/healthcare13131610 (PMC12250147; doi:10.3390/healthcare13131610)
Supplement: Supplementary file 1 [file healthcare-13-01610-s001.zip › healthcare-3698948-supplementary.pdf]

Supplementary material I: Statistical Analysis and Robustness checks

A Repeated Measured Multivariate of CoVariance (RM-MANCOVA) is a sophisticated general linear model that combines an Analysis of Variance (ANOVA) component and a regression-based component. One the advantages of this statistical analysis is that it allows for the creation of a linear model that incorporates multiple dependent variables and both between-subject and within-subject factors. Moreover, the regression-based component of an RM-MANCOVA allows for the derivation of estimated means after isolating the effects of one or more control variables. Therefore, it should be preferred to linear regression models when multiple variables are of interest and when prior studies suggest that said variables might covary systematically (be correlated). Moreover, the RM-MANCOVA allows for the incorporation of the effects of time between each data point.

Following the above logic, we constructed a general linear model that included HR, dyspnoea, and SpO_2 as dependent variables. Scores on the 6MWT-D, PPT, VAS-HN, VAS-S, VAS-D, VAS-L, VAS-PH, SARC-F, FRAIL, and EMI were covariates. Physical activity level was a between-subject predictor, and time elapsed between measurements was a within-subject factor. An interaction term tested whether variations in measurements were influenced by physical activity level. For RM-MANCOVA results, a non-significant Box's M test would ensure the trustworthiness of a multivariate solution (indicating that the assumption of the homogeneity of covariances was met). Similarly, Mauchly's W is a statistic that test for sphericity, meaning that the variances across all possible pairs of within-subject conditions have homogenous variances. However, this test requires three datapoints to be conducted.

Significant p-values for the F-test of either Wilks' Lambda statistic or Pillai's trace indicate predictor effects on dependent variables. A significant between–within interaction term suggests that the effects of elapsed time on dependent variables vary across physical activity levels. Partial Eta squared (partial η2) can be converted into Cohen's d effect sizes.

Robustness checks

Table 1 shows that, as expected, our dependent variables were significantly correlated (See Appendix 1, Table 1). Thus, employing an RM-MANCOVA approach to estimate marginal means is justified. More precisely, pre-test scores of heart rate and dyspnoea were positively correlated (r = .22**), but that was not the case for pre-test scores of heart rate and SpO2. Yet, all post-test scores for all three metrics were correlated with each other, indicating the need to adopt a multivariate approach. On the other hand, pre-test and post-test scores of heart rate (r = .67**), dyspnoea (r = .53***), and SpO2 (r = .30***) were significantly correlated, indicating the need to incorporate a within-subject factor into the linear model.

Box's M is a test based on the chi-square test. It is not without its limitations[64]. For example, some authors argue that Box's M test is sensitive to (a) departure from normality and (b) sample size, meaning that samples that are either too small or too large might affect the test's results. In those cases when the RM-MANCOVA model does not pass Box's M test, several authors recommend either using Pillai's trace, a statistic that is more robust to violations of assumptions on linear models[65], or, as an alternative, using a more conservative approach for testing effects, that is, employing univariate analyses for each dependent variable. Regarding the sphericity assumption, given that we only had two data points, we did not need to correct for the effects of potential violations of the sphericity assumption when testing the effect of elapsed time between measurements (pre-test vs. post-test scores).

Box's M test for our multivariate solution was significant (F (42, 68368.56) = 4.18 p <.001.). Therefore, in the present study, we report the univariate solutions for our dependent variables (HR, SpO_2, and dyspnoea), adopting a more conservative approach to the interpretation of our findings. However, as supplementary information, Appendix 1, Table 2, shows the multivariate solutions with both Wiks Lambda and Pillai's trace values.

Control Variables

We detected significant between-subjects differences across PA levels in HR, dyspnoea, and SpO_2 in our sample (see Table 3, Appendix 1, Table 2). Regarding HR, the analysis showed that the covariates 6MWT-D and sarcopenia had an effect on HR scores. The first covariate, 6MWT-D, had a medium-to-large effect (F (1, 148) = 15.66; p <.001; partial η2 = .10; Cohen's d = .65), whereas sarcopenia had a small-to-medium effect on HR (F (1, 148) = 5.10; p <.05; partial η2 = .03; Cohen's d = .36). In relation to dyspnoea, PPT and two facets of the exercise motivation index (social and psychological) had an effect on its values. PPT had a moderate-to-large effect on dyspnoea (F (1, 148) = 8.10; p <.01; partial η2 = .05; Cohen's d = .47). Similarly, social motivation (F (1, 148) = 13.72; p <.001; partial η2 = .09;

Cohen's d = .61) and psychological motivation ($F(1, 148) = 5.50$; $p < .05$; partial $\eta 2 = .04$; Cohen's d = .39) had small-to-moderate effects on participants' dyspnoea. Finally, none of our covariates had an effect on SpO_2. However, we detected a medium-to-large effect of the level of PA on SpO_2 ($F(1, 148) = 8.47$; $p < .0001$; partial $\eta 2 = .10$; Cohen's d = .68).

Appendix 1, Table 1. Pearson's product–moment correlation for all variables in the study.

	MEAN	SD	1.	2.	3.	4.	5.	6.	7.	8.	9.	10.	11.	12.	.13	.14	.15	.16	.17	.18
**1. 6MWT-D**	506.49	66.6	-																	
**2. EMIPHYS**	3.45	.64	.13	-																
**3. EMIPSYCH**	3.25	.63	.07	**.64****	-															
**4. EMI - SOC**	2.94	.97	.15	**.50****	**.61****	-														
**5. VAS-HN**	2.34	2.76	-.08	.04	-.02	-.20*	-													
**6. VAS – S**	1.79	2.79	-.13	.03	.002	-.04	.43**	-												
**7. VAS – D**	1.80	2.07	.03	-.15	-.06	-.18*	.39**	.26**	-											
**8. VAS– L**	3.67	3.14	-.23**	-.11	-.11	-.12	.22**	.08	.19**	-										
**9. VAS- PH**	2.17	3.00	-.11	.05	-.07	-.12	.12	.25*	.21**	.02	-									
**10. PPT (MEAN)**	3.45	1.38	.11	.05	.07	-07	-.05	-.12	.03	.14	-.09	-								
**11. FRAILTY**	.27	.52	-.20**	-.11	-.06	-.08	.03	.002	.07	.22**	.12	-.05	-							
**12. SARC**	.01	.11	.05	-.25**	-.08	-.05	-.10	-.01	-.06	.01	-.06	-.06	.16**	-						
**13. A. LEVEL**	3.08	.84	-.04	-.02	-.01	-.11	-.09	-.09	.04	.11	.02	.10	-.02	-.14.	-					
**14. HR– PRE**	78.08	12.08	.21**	.12	.02	.13	-.08	-.14	-.21*	-.13	-.19*	.03	-.08	-.12	-.12	-				
**15. HR– POST**	94.17	21.07	.39**	.21**	.05	.28**	-.12	-.13	-.22*	-.23**	-.17*	-.10	-.07	-.11	-.35**	.67**	-			
**16. DYS- PRE**	.52	.82	.16*	.08	.06	.27**	-.13	.05	-.09	-.15	.08	-.29**	.06	-.003	-.22**	.22**	.36**	-		
**17. DYS-POST**	1.37	1.78	.05	.20**	.06	.30**	.10	.19*	.04	-.07	.03	-.23**	.02	-.02	-.36**	.17**	.33**	.53***	-	
**18. SPO_2 – PRE**	97.40	1.36	.13	-.06	.05	-.05	.02	-.02	.09	.03	-.07	.03	-.01	.05	.15	-.09	-.18*	-.06	-.05	-
**19. SPO_2-POST**	96.70	2.43	-.10	-.10	-.09	.18*	.19*	.13	.21**	.20*	.10	.12	.08	.05	.28**	-.30**	-.40**	-.25**	-.20**	.30**

*Note: ** p <.01; * p <.05; SD: standard deviation;* 6MWT-D*: 6-minute walk distance; EMI – Phys = motivation to exercise—physical; EMI-Psych = motivation to exercise—psychological; EMI-Soc = motivation to exercise—social; VAS-HN* = Visual Analogue Scale, Head and Neck; VAS – S= Visual Analogue Scale, Shoulder; VAS-D= Visual Analogue Scale, Dorsal; VAS-L= Visual Analogue Scale, Lumbar; VAS-PH= Visual Analogue Scale, Pelvis and Hip; PPT= pressure pain threshold ; SARC = *sarcopenia;* A**.** LEVEL= activity level; *HR = heart rate;* DYS= dyspnoea; *SPO2 = oxygen saturation.*

Appendix I, Table 2

Repeated-measures ANCOVA for the effect of physical activity levels on heart rate, dyspnoea, and oxygen saturation.

Between-Subject Effects (BS)	*Statistic*	*F-Value*	*Effect Size*
Physical Activity Group – PA	Wilk's λ = .79	F (6, 292) = 6.20, p <.001	Partial η2 = .11; Cohen's d = .71
	Pillai's Trace = .22	F (6, 292) = 5.93, p <.001	Partial η2 = .11; Cohen's d = .70
Within-Subject Effects (WS)			
Elapsed Time	Wilk's λ = .97	F (3, 146) = 1.34, ns	Partial η2 = .03; Cohen's d = .35
	Pillai's Trace = .03		
WS x Control Variables			
Elapsed Time x 6MWT-D	Wilk's λ = .87	F (3, 146) = 7.44, p <.001	Partial η2 = .13; Cohen's d = .77
	Pillai's Trace = .13		
Elapsed Time x EMI – Phys	Wilk's λ = .95	F (3, 146) = 2.72, p <.05	Partial η2 = .05; Cohen's d = .46
	Pillai's Trace = .05		
Elapsed Time x EMI – Soc	Wilk's λ = .92	F (3, 146) = 4.16, p <.01	Partial η2 = .08; Cohen's d = .59
	Pillai's Trace = .08		
Elapsed Time x EMI – Psych	Wilk's λ = .92	F (3, 146) = 4.20, p <.01	Partial η2 = .08; Cohen's d = .59
	Pillai's Trace = .08		
Elapsed Time x VAS-HN	Wilk's λ = .96	F (3, 146) = 1.83, ns	Partial η2 = .04; Cohen's d = .41
	Pillai's Trace = .04		
Elapsed Time x VAS – S	Wilk's λ = .99	F (3, 146) = .47, ns	Partial η2 = .01; Cohen's d = .20
	Pillai's Trace = .01		
Elapsed Time x VAS – D	Wilk's λ = .99	F (3, 146) = .76, ns	Partial η2 = .02; Cohen's d = .29
	Pillai's Trace = .02		
Elapsed Time x VAS– L	Wilk's λ = .99	F (3, 146) = .64, ns	Partial η2 = .01; Cohen's d = .20
	Pillai's Trace = .01		
Elapsed Time x VAS- PH	Wilk's λ = .97	F (3, 146) = 1.43, ns	Partial η2 = .03; Cohen's d = .35
	Pillai's Trace = .03		
Elapsed Time x PPT	Wilk's λ = .96	F (3, 146) = 2.03, ns	Partial η2 = .04; Cohen's d = .41
	Pillai's Trace = .04		
Elapsed Time x FRAILTY	Wilk's λ = .99	F (3, 146) = .41, ns	Partial η2 = .01; Cohen's d = .20
	Pillai's Trace = .01		
Elapsed Time x SARCOPENIA	Wilk's λ = .98	F (3, 146) = 1.11, ns	Partial η2 = .02; Cohen's d = .29
	Pillai's Trace = .02		
Between–Within-Subject Effect			
Elapsed Time x PA	Wilk's λ = .75	F (6, 292) = 7.60, p <.001	Partial η2 = .14; Cohen's d = .81
	Pillai's Trace = .26	F (6, 294) = 7.18, p <.001	Partial η2 = .13; Cohen's d = .77

Note: 6MWT-D*: 6-minute walk distance; EMI – Phys = motivation to exercise—physical; EMI-Psych = motivation to exercise—psychological; EMI-Soc = motivation to exercise—Social; VAS-HN* = Visual Analogue Scale, Head and Neck; VAS – S= Visual Analogue Scale, Shoulder; VAS-D= Visual Analogue Scale, Dorsal; VAS-L= Visual Analogue Scale, Lumbar; VAS-PH= Visual Analogue Scale, Pelvis and Hip; PPT= pressure pain threshold; ns = non-significant.
